# Real-world experience with selumetinib in children with neurofibromatosis type 1: a multicentric retrospective study

**DOI:** 10.1007/s11060-025-05197-5

**Published:** 2025-08-13

**Authors:** Claudia Santoro, Mariachiara Servedio, Maria Cristina Diana, Irene Russo, Elena Arkhangelskaya, Gianluca Piccolo, Andrea Santangelo, Angela Mastronuzzi, Antonella Cacchione, May El Hachem, Carmela Russo, Mario Cirillo, Ilaria Cecconi, Antonio Grasso, Mariateresa Loiotine, Nicola Santoro, Mariachiara Resta, Carmela De Meco, Consolata Soddu, Eugenia Spreafico, Bartolomeo Rossi, Chiara Fossati, Chiara Leoni, Silverio Perrotta, Teresa Perillo

**Affiliations:** 1 Department of Women’s and Children’s Health and General and Specialized Surgery, University of Campania “Luigi Vanvitelli”, Naples, Italy; 2 Division of Pediatric Oncology and Haematology “Policlinico di Bari”, Bari, Italy; 3 Pediatric Neurology and Neuromuscular Disorders Unit, IRCCS Istituto Giannina Gaslini, Genova, Italy; 4 Radiology Unit, IRCCS Istituto Giannina Gaslini, Genova, Italy; 5 Neuro-Oncology Unit, IRCCS Istituto Giannina Gaslini, Genova, Italy; 6 Università degli Studi di Genova, Genova, Italy; 7 Onco-Hematology, Cell Therapy, Gene Therapies and Hemopoietic Transplant Unit, “Bambino Gesù Children’s Hospital”, IRCCS, Rome, Italy; 8 Dermatology Unit and Genodermatosis Research Unit, “Bambino Gesù Children’s Hospital”, IRCCS, Rome, Italy; 9 Neuroradiology Unit, Department of Neurosciences, “Santobono- Pausilipon Children’s Hospital”, Naples, Italy; 10 Neuroradiology Unit, Department of Advanced Medical and Surgical Sciences, University of Campania “Luigi Vanvitelli”, Naples, Italy; 11 Unit of Child Neuropsychiatry, IRCCS Azienda Ospedaliero-Universitaria di Bologna, “Policlinico Sant’Orsola”, Bologna, Italy; 12 Pediatric Hematology and Oncology, IRCCS Azienda Ospedaliero- Universitaria di Bologna, “Policlinico Sant’Orsola”, Bologna, Italy; 13 Division of Neuroradiology, Policlinico di Bari, University Aldo Moro of Bari, Bari, Italy; 14 “Fondazione Casa Sollievo della Sofferenza-Opera di San Pio da Pietralcina, San Giovanni Rotondo”, Foggia, Italy; 15 Pediatric Clinic and Rare Diseases, Microcitemico Hospital “A. Cao”, University of Cagliari, Cagliari, Italy; 16 Pediatric Clinic, Division of Pediatric Neurology and Electroencephalography - IRCCS Foundation, Policlinico San Matteo, Università degli Studi di Pavia, Pavia, Italy; 17 Hematology Oncology Division, Department of Women’s and Children’s Health, University of Padova, Padua, Italy; 18 IRCCS San Gerardo dei Tintori, Pediatria, Monza, Italy; 19 Center for Rare Diseases and Birth Defects, Department of Woman and Child Health and Public Health, Fondazione Policlinico Universitario A. Gemelli, IRCCS, Rome, Italy

**Keywords:** Neurofibromatosis type 1, Plexiform neurofibroma, Selumetinib, Clinical benefit rate, Safety, Quality of life

## Abstract

**Purpose:**

Selumetinib is a MEK inhibitor indicated for pediatric patients with neurofibromatosis type 1 (NF1) and symptomatic inoperable plexiform neurofibromas (PNs).

**Methods:**

This retrospective study collected data from 70 patients (aged 3 − 18 years) with NF1 and symptomatic inoperable PNs treated with selumetinib as part of compassionate use at 11 Italian centers between October 2018 and October 2024. Assessments included the clinical benefit rate (CBR) after 24 months and at the last observation. Major response (MR) was defined as a ≥ 50% reduction from baseline in tumor volume. Adverse events (AEs), patient-reported pain and quality of life (QoL), and Eastern Cooperative Oncology Group performance status (ECOG PS), were also evaluated.

**Results:**

Of 45/70 patients with available natural history data at C0, 33/45 (73.3%) had progressive disease (PD). Radiological evaluation at C6 in 17/33 patients showed 16 (94.1%) had stable disease (SD) or partial response (PR). 52/58 patients (91.5%) had SD or PR/MR at C12; final response at last radiological follow-up was PD (7.7%), SD (42.3%), PR (30.8%) and MR (19.2%). CBR was 83.3% (24/70) at C24 and 91.5% (43/47) at last radiological follow-up. Selumetinib significantly reduced pain perception and improved QoL and ECOG PS. The type of response at C6 seems to predict response at C12 and at last observation. Adverse events were generally mild (78% grade ≤ 2).

**Conclusion:**

Our findings suggest that the response after 6 and 12 selumetinib cycles may predict long-term outcomes, providing clinicians with an early indicator for therapeutic decision-making.

**Trial registration number:**

Not applicable.

**Supplementary Information:**

The online version contains supplementary material available at 10.1007/s11060-025-05197-5.

## Introduction

Neurofibromatosis type 1 (NF1) is a multisystemic, autosomal dominant neurocutaneous disorder that affects ∼1 in 3000 individuals worldwide [1]. Plexiform neurofibromas (PNs) are one of the most distinctive signs of NF1, occurring in 30 − 50% of cases [2]. They originate from Schwann cells and can grow throughout life; their growth rate is particularly high during the first decade of life [2, 3].

PNs can cause a large variety of signs and symptoms that are related, in part, to their volume, as well as their compression on nearby organs and anatomic structures [4]. PNs frequently cause pain and disfigurement, significantly impacting quality of life (QoL) [4, 5]. Although PNs can be often surgically treated, including through debulking procedures, the risk of iatrogenic complications and recurrence remains high [4]. This is particularly the case in early childhood, when tumor regrowth is more likely due to the natural history of the disease [3].

Selumetinib is a selective MEK1/2 protein inhibitor [6] that was approved in 2020 by the United States (US) FDA, and in 2021 by the European Medicines Agency (EMA), for the treatment of children with NF1 and symptomatic, inoperable PNs [7, 8]. In oncology, the clinical benefit rate (CBR) is defined as the total number (or percentage) of patients who achieve complete response, partial response (PR), or disease stabilization for ≥ 6 months as a result of therapeutic intervention [9]. Current evidence indicates that PNs do not undergo complete radiologic regression with selumetinib [5, 10]. However, the drug effectively prolongs progression-free survival (PFS) and stabilizes tumor growth, preventing the onset or worsening of symptoms [5, 10]. We therefore focused on CBR as a more important outcome than simply radiologic tumor shrinkage, and explored symptom regression even in cases of radiologic stable disease (SD). This study aimed to fill the gap created by the paucity of real-world data and the limitations of using just radiological response criteria in these patients.

## Materials and methods

### Study overview

This was a non-profit, observational, retrospective, multicenter study, approved by the Ethics Committee of the Polyclinic of Bari, Italy, on April 24, 2024. Patients were treated with selumetinib under a compassionate use approved in 11 Italian sites.

The study was conducted in accordance with the Helsinki Declaration, Good Clinical Practice guidelines, and all applicable laws and regulations. Written informed consent to participate in the compassionate use access and Data Protection Impact Assessment (DPIA) forms were completed by or on behalf of all participants.

### Objectives

The primary objective of this study was to report the growth rate of the target PN during the first year of treatment, seeking any predictive factors with respect to the final outcome in Italian pediatric patients (aged 3 − 18 years) with NF1 and inoperable, symptomatic PNs treated with selumetinib. We also calculated the clinical benefit rate (CBR) at the last observation. We introduced the major response criteria into the CBR: achievement of a volume reduction of 50% or more compared to the basal value. We also evaluated at 6 cycles the clinical benefit in those patients with radiological SD.

The secondary objectives included evaluating the safety profile of selumetinib and investigating the impact of therapy on secondary morbidities, specifically QoL, pain, and Eastern Cooperative Oncology Group performance status (ECOG PS), which describes a patient’s level of functioning in terms of their ability to care for themself, daily activity, and physical ability (walking, working, etc.). The ECOG PS numbering scale is graded 0 − 5; a value of 0 indicates that a patient is fully active and able to engage in all pre-disease performance without restriction. The exploratory endpoints included the effect of therapy on other secondary morbidities, namely disfigurement/deformity, obstruction of upper airways, and motor impairment, using standardized tests performed at multiple timepoints.

### Study population

Patients aged 3 − 18 years with a clinical diagnosis of NF1 according to revised criteria [11], with symptomatic and inoperable PNs, and who received selumetinib at a dosage of 25 mg/m^2^ twice a day for ≥ 6 cycles were enrolled in the study, only if their PNs could be evaluated through the established recommendations of the Response Evaluation in Neurofibromatosis and Schwannomatosis (REiNS) Collaboration [12]. Exclusion criteria included prior treatment with other MEK inhibitors within 3 months of selumetinib initiation, and incomplete radiologic data preventing adequate tumor evaluation.

### Safety and modification of therapy

Adverse events (AEs) were classified according to Common Terminology Criteria of Adverse Events (CTCAE version 5.0) and managed according to Good Clinical Practice guidelines. Dose reductions and temporary suspensions of selumetinib were registered, as well as their causes.

### Evaluations

The following patient data were retrospectively collected: demographic information; age at NF1 and PN diagnosis; anatomical location of PNs; history of surgical resection or biopsy before selumetinib initiation; start and end dates of selumetinib therapy; symptoms and clinical indications for treatment; PN volume at baseline and at predefined intervals (every 6 cycles until the last observation performed for each patient); reasons for treatment discontinuation; and AEs observed during therapy. The collection of data on assessments was part of the routine data collection. Measures were calculated with local 3-diameter software and reported by site.

To ensure consistency and clarity, the timepoints in this study are denoted by the letter ‘C’ followed by the corresponding treatment cycle number (e.g., C6, C12, C24), to prevent ambiguity with other temporal conventions and allow a direct correlation between clinical and radiologic assessments and treatment progression. Available data that fell in any of the specified timepoints, every 6 ± 1 cycle, were collected.

Retrospective, standardized clinical evaluations, using standardized and repeatable functional or instrumental tests, were performed for the assessment of the exploratory endpoints.

### Response criteria

Treatment response was evaluated based on magnetic resonance imaging (MRI) volumetric tumor assessments at C6, C12, C24, and at last observation. The following response criteria were applied: PR, a ≥ 20% reduction from baseline in tumor volume, maintained for ≥ 4 weeks; MR, PR with a ≥ 50% reduction in tumor volume; SD, a change from baseline of < 20% in tumor volume; progressive disease (PD), a ≥ 20% increase from baseline in tumor volume; and CBR, the proportion of patients achieving a PR, MR, or SD after ≥ 24 cycles of treatment [13].

Pain was evaluated by the Numeric Rating Scale (NRS) [14] or the Visual Analog Scale (VAS) [15]; health-related QoL was evaluated using the Pediatric Quality of Life Inventory (PedsQL) questionnaire [16]; and functional status and ability to perform daily activities were evaluated using the ECOG PS scale [17].

### Statistical analysis

A Pearson’s correlation analysis with Bonferroni correction was performed to evaluate correlations between the radiological response at different timepoints (C6, C12, and at the last visit) and other variables (i.e., age at the start of therapy, surgery, previous pharmacotherapies, the duration of selumetinib therapy, and the occurrence of AEs). Statistical analysis was performed using SPSS software (IBM SPSS Statistics for Windows, version 20; IBM Corp., Armonk, NY, USA). The p-value associated with the Pearson correlation was corrected by Bonferroni’s correction and set at 0.0023. The paired Wilcoxon signed-rank test was used to compare VAS scores and ECOG PS between baseline) and follow-up timepoints (C6, C12, C18, C24, until C48). For this analysis, *p* < 0.05 was considered statistically significant.

## Results

### Study population

Of the 70 enrolled patients, 10 of whom were previously described in an Italian study [18], the median age at NF1 diagnosis was 1 year and the median age at PN diagnosis was 6 years (Table 1). Radiological follow-up of previous treatment was available only for 45/70 patients; 73% (33/45) had confirmed disease progression at the beginning of treatment. At study closure, the median patient age was 15.4 years, and the median number of treatment cycles was 49.

**Table 1:** Baseline patient demographic and clinical characteristics of the study population

Characteristic	All patients (*N* = 70)
Sex, *n* (%)	
Male	39 (55.7)
Female	31 (44.3)
Age at NF1 diagnosis, median (range), years	1 (0–12)
Age at PN diagnosis, median (range), years	6 (0–15)
Age at therapy start, median (range), years	7.9 (2.6–12.6)
Age at the closing date of the study, median (range), years	15.4 (5.6–22.7)
PN volume at baseline, median (range), mL	126.8 (5–3820)
Number of treatment cycles, median (range)	49 (7.5–79)
Location of the target PN, *n* (%)	
Head	19 (27.1)
Neck	12 (17.1)
Lower abdomen	12 (17.1)
Mediastinum/thorax	7 (10.0)
Lower legs	5 (7.1)
Upper arm	3 (4.3)
Upper abdomen	3 (4.3)
Thigh	3 (4.3)
Hand	2 (2.9)
Foot	2 (2.9)
Forearm	1 (1.4)
Ankle	1 (1.4)
Signs and symptoms related to target PN, *n* (%)	
Pain	36 (51.4)
Deformity	25 (35.7)
Motor dysfunction	11 (15.7)
Compression of neck vessels	3 (4.3)
Dysphagia	3 (4.3)
Paresthesia	3 (4.3)
Rachialgia	3 (4.3)
Visual deficit	3 (4.3)
Dysarthria	2 (2.9)
Incontinence	2 (2.9)
Limp	2 (2.9)
Ptosis	2 (2.9)
Pubalgia, dyspnea	2 (2.9)
Hearing loss	1 (1.4)
Hyposthenia of upper limb	1 (1.4)
Hypotrophy of upper limb	1 (1.4)
Overlying skin redness	1 (1.4)
Paralysis of upper limb	1 (1.4)
Therapy suspension, *n* (%)	12 (17.1)
Poor treatment compliance	3 (2.5)
Grade 4 AEs	2 (17.0)
MPNST progression	2 (17.0)
Radiologic progression	2 (17.0)
AEs and resolution of PN-related symptoms	1 (8.3)
Medication holiday	1 (8.3)
Symptom progression	1 (8.3)

AEs were classified according to CTCAE v5.0

AEs, adverse events; CTCAE v5.0, Common Terminology Criteria for Adverse Events version 5.0; MPNST, malignant peripheral nerve sheath tumor; NF1, neurofibromatosis type 1; PN, plexiform neurofibromas

**Fig. 1:**
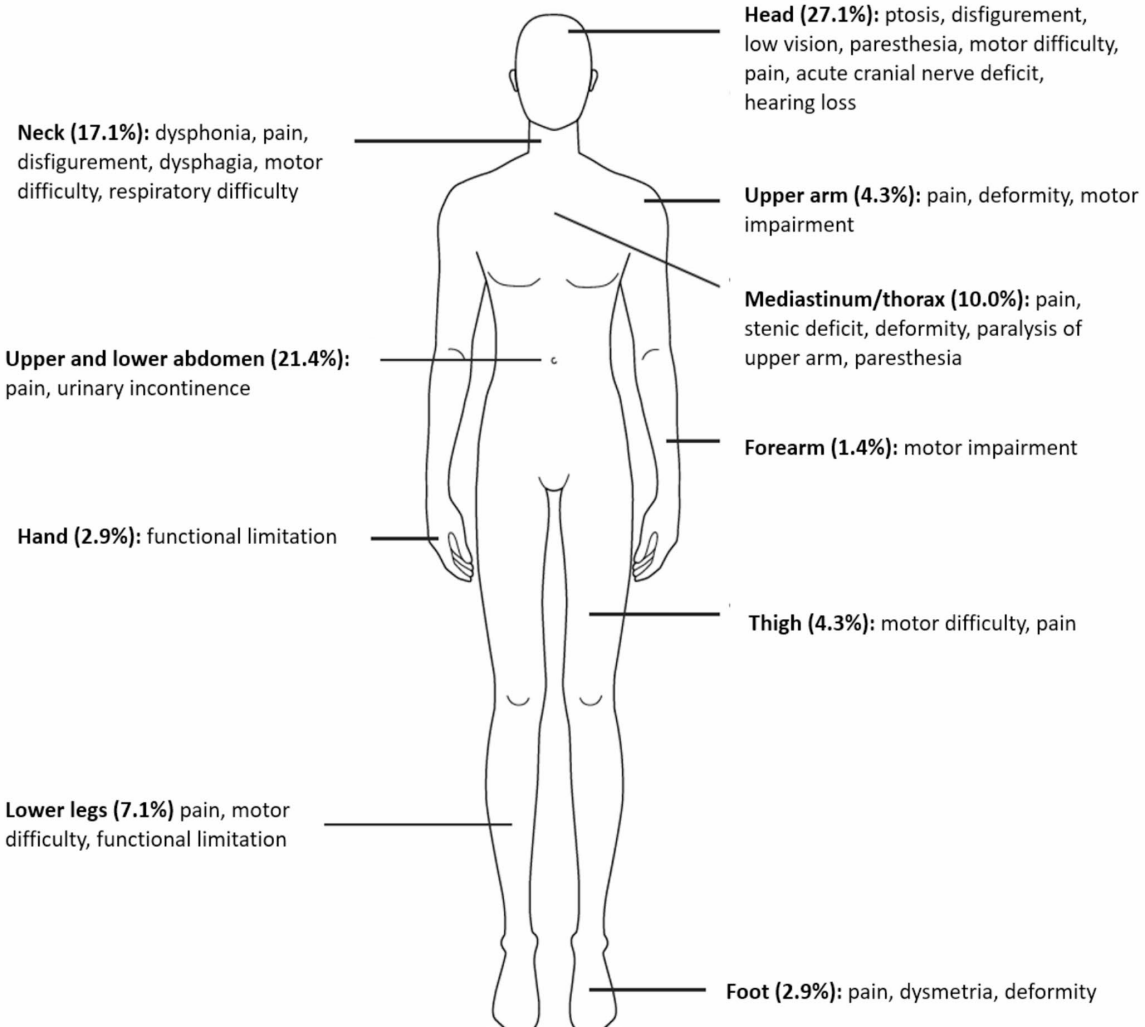
Graphical representation of symptoms and signs of plexiform neurofibromas with their frequency and anatomical location of causative plexiform neurofibromas

PNs were primarily located in the head (27.14%), neck (17.14%), upper and lower abdomen (21.42%), and the mediastinum/thorax (10.00%; Fig. 1). Seventeen patients (24.3%) had two clinical symptoms or signs at baseline. The most frequent symptoms were pain (51.4%), deformity (35.7%), and motor dysfunction (15.7%).

Selumetinib was suspended in 12 patients due to poor treatment compliance (*n* = 3), grade 4 AEs (*n* = 2, paronychia and skin toxicity in 1 patient each), malignant peripheral nerve sheath tumor (MPNST) progression (*n* = 2), radiologic progression (*n* = 2), AEs and resolution of PN-related symptoms (*n* = 1), medication holiday (*n* = 1), and symptom progression (*n* = 1).

Four patients had received other therapies prior to initiating selumetinib: sirolimus (*n* = 1), pegylated interferon (*n* = 1), and imatinib followed by pegylated interferon (*n* = 2). Nineteen patients had undergone surgical procedures consisting of functional debulking (*n* = 14) and biopsy for suspected malignant evolution (*n* = 5).

### Effectiveness and safety outcomes

#### Radiological response

Data on the natural history of the target PN prior to therapy was available in 45 of the 70 enrolled patients. Of these, 33/45 patients had PD at C0. An early radiological evaluation at C6 was available in 17 of the 33 patients, and 16/17 (94.1%) had a SD or PR.

Independently by the natural history of the target PN prior to therapy, an MRI assessment was available at C6 for 47/70 patients (67.1%; Supplementary Figure S1). Among them, 29 patients (61.7%) had SD and 16 (34.0%) PR/MR (Table 2). At the last radiologic follow-up, the CBR rate among these 47 patients was 91.5%.

**Table 2:** Radiological response after 6 and 12 treatment cycles, and at the end of follow-up

	PR	MR	SD	PD
C6 (47 patients), n (%)	16 (34.0)	0	29 (61.7)	2 (4.3)
Median variation, % of volume (range)	−34.6 (–20.2, − 49.5)	–	–	+ 29.1 (+ 20.5, + 245.8)
C12 (58 patients), n (%)	13 (22.4)	8 (13.8)	31 (53.4)	6 (10.3)
Median variation, % of volume (range)	−35.3 (− 30.3, − 49.1)	−57.27 (− 50.1, 76.6)	–	+ 44.35 (+ 20.5, + 245.8)
End of follow-up (70 patients), n (%)	21 (30.0)	11 (15.7)	29 (41.4)	9 (12.9)
Median variation, % of volume (range)	−33.2 (–20.8, − 47.6)	−63.3 (–50.3, − 94.0)	–	+ 55 (+ 31.7, + 323.7)

C, cycle; MR, major response; PD, progressive disease; PR, partial response; SD, stable disease

Of 52/58 patients (90.0%) who achieved SD or PR/MR at C12, the final response, measured at the last radiologic follow-up, was: PD in 4 (7.7%), SD in 22 (42.3%), and PR in 16 (30.8%) children; 10 patients (19.2%) achieved MR.

MRI data at C24 was available in 24 of the 70 patients. Ten (41.6%) had SD (median variation of volume − 1.4 [range − 13.4 to + 11.4]), 10 had a PR/MR (median variation of volume − 59.8 [range − 30 to -93]), of which 6/10 was a MR, and 4/10 PD (median variation of volume + 57.1 [range + 31.7 to + 78.1]). Therefore, a CBR at C24 was observed in 83.3% of cases.

Radiological responses at C6, C12, and at the end of follow-up are presented in Table 2, along with median changes in tumor volume for PR and MR.

Data regarding four females and five males with PD at the last observation are reported in Supplementary Tables 1 and 2. Four patients had undergone a biopsy prior to initiating selumetinib, two of whom are deceased and have been cited in a previous publication [18]. The median duration of treatment was 24 cycles (range: 18 − 42).

Among all patients, independently of timepoint, the best MR was observed at C24 and equaled 94% of reduction from the baseline volume.

Supplementary Figure S2 shows trends over time in percentage changes of target PN volumes for individual patients.

#### Early clinical benefit

Twenty-nine patients were found to have SD at C6, of whom 12 (41.4%) had pain at C0. At C6, 9/12 (75.0%) had complete pain regression, and pain remained stable in the other 3 patients.

#### ECOG PS

Among 57 patients (81.4%) with available baseline ECOG PS values, the average value was 0.7, the median 0 (range: 0 − 3). At C6, further evaluations for 20 (35.1%) of these 57 patients revealed an average ECOG PS of 0.3 (range: 0 − 2); at C12, the value was 0 in 14 of the 57 patients with available data.

We further considered ECOG values at all time points (C6 through C36), for the whole dataset. ECOG PS was significantly improved from baseline with selumetinib treatment (*p* < 0.05; Table 3), as demonstrated independently by radiological response.

**Table 3:** Changes from baseline in ECOG PS and VAS pain scale scores after selumetinib treatment

	C6	C12	C18	C24	C30	C36	C42^c^	C48^c^
**ECOG PS**	*N* = 40/57	N 37/57	*N* = 27/57	*N* = 17/57	*N* = 11/57	*N* = 22/57		
*** Z-*** **score** ^**a**^	−3.274	−3.418	−1.997	−3.030	−2.280	−2.121	NA	NA
** P-value** ^**b**^	0.001	0.001	0.046	0.002	0.023	0.034	NA	NA
** Median value (range)**	1 (0–2)	0 (0–2)	1 (0–2)	1 0–2)	1 (0 − 2)	0 (0 − 2)	0	0
**VAS pain scale**	*N* = 44/60	*N* = 39/60	*N* = 31/60	*N* = 19/60	*N* = 11/60	*N* = 21/60	*N* = 6/60	*N* = 5/60
*** Z-*** **score** ^**a**^	−3.931	−3.606	−2.871	−2.255	−2.207	−2.023	−1.342	−1.342
** P-value** ^**b**^	0.000	0.000	0.004	0.004	0.027	0.043	0.180	0.180
** Median change (range)**	1.4 (0–6.5)	1.8 (0–6)	1.3 (0–9)	0.8 (0–9)	0.2 (0 − 2)	0.1 (0 − 2)	0	0

^a^Wilcoxon test values based on positive ranking

^b^Asymptomatic significance 2-tailed p-value

^c^ECOG values were available just for a very few patients at C42 and C48 (7 patients with a median value of 1 (0–1), and 6 patients with a median value of 0. (0–1) respectively. Thus, they were not considered for statistical analysis

C, cycle; ECOG PS, Eastern Cooperative Oncology Group performance status; NA, not available; VAS, visual analog scale

ECOG was available only for a few patients at C42 and C48 (7 patients with a median value of 1 (0–1), and 6 subjects with a median value of 0. (0–1) respectively) thus they were not considered for statistical analysis. At 66 cycle 5 patients still on therapy were evaluated and all had ECOG 0 values.

#### Pain

Pain perception was assessed in 33/36 patients (91.7%) presenting with pain at baseline, although 60 patients in total were administered the VAS pain scale. Pain perception significantly decreased from baseline at all timepoints (C6 − C48; *p* < 0.05 for all comparisons; Table 3), independently by radiological response. At C12, VAS scores ranged from 0 − 6 (mean 1.2), versus 0 − 9 (mean 2.3) at baseline, indicating a clear improvement. Median changes from baseline in VAS pain scores from C6 − C48 are presented in Table 3. The perception of pain at the start of therapy was significantly related to a longer duration of therapy (*p* = 0.000).

#### QoL

Eighteen patients (25.7%) completed the PedsQL at baseline and all assessed timepoints. At baseline, the mean PedsQL score in these patients was 40 (on a scale of 0–100, with higher scores indicating poorer QoL), which improved to 19 at a median of 12 cycles (range: 6–48), independently according to the radiological response. Specifically, 15 patients reported an improvement in PedsQL scores during selumetinib treatment, two patients had no change, and QoL worsened in one patient. The PedsQL questionnaire was also administered to seven parents. At baseline, the mean PedsQL score reported among these parents was 34, which improved to 22 at the last observation (median of 12 cycles [range: 12–36]).

#### Selumetinib-related toxicity

Selumetinib-related toxicity data were available for 61 patients (87.1%). At least one toxicity possibly related to drug safety was detected in 49 (80.3%) of those patients. A total of 21 patients had two AEs, 9 had three AEs, 3 had four AEs, and 16 had just one AE. Three patients discontinued selumetinib due to AEs (two cases of grade 4 paronychia, and one case of recurrent grade 3 creatine phosphokinase [CPK] elevation).

A total of 91 AEs were observed, the most common of which were maculopapular rash (37.4% of events), paronychia (30.8%), and xerosis with/without itching (14.3%). Most (78.0%) AEs were grade ≤ 2 in intensity. Table 4 presents the distribution of AEs by severity level during selumetinib treatment.

**Table 4:** Number, type, and grade of adverse events reported for 61 patients during selumetinib treatment

	Grade 1 and 2 (*n* = 71)	Grade 3 (*n* = 18)	Grade 4 (*n* = 2)	Total AEs (*n* = 91)
Number of events, (%)				
Dermatitis	28 (39.4)	6 (33.3)		34 (37.4)
Paronychia	18 (25.4)	8 (44.4)	2 (100.0)	28 (30.8)
Xerosis ± itching	13 (18.3)			13 (14.3)
CPK level increase	4 (5.6)	3 (16.7)		7 (7.7)
Hair depigmentation	4 (5.6)			4 (4.4)
Abdominal pain	1 (1.4)			1 (1.1)
Nausea and vomiting	1 (1.4)			1 (1.1)
Diarrhea	1 (1.4)			1 (1.1)
Eyelid edema	1 (1.4)			1 (1.1)
ALT/AST transaminase ratio increase		1 (5.6)		1 (1.1)
CPK level increase, rhabdomyolysis and regression			1 (50.0)	1 (1.1)

AEs were evaluated using the Common Terminology Criteria for Adverse Events (CTCAE) version 5.0

ALT/AST, alanine transaminase/aspartate transaminase; CPK, creatinine phosphokinase

The median time to AE onset after selumetinib initiation was 6.1 months (range: 0.5 − 40). Approximately one-third of all AEs were observed very early, within the first month of therapy, and 76.9% were observed within 6 months of therapy.

### Exploratory outcomes

#### PN-related symptoms

Five patients reported a total of nine PN-related symptoms and signs: disfigurement/deformity (*n* = 4); obstruction of upper airways (*n* = 1); dysphonia (*n* = 1); and motor impairment (*n* = 3). These were measured during selumetinib treatment with the modified Ablon scale (for disfigurement/deformity) [19], the manual muscle test (MMT, for motor impairment) [20], the functional ambulation categories (FAC) test (for motor impairment) [21], voice quality rated by the grade (overall grade of hoarseness), roughness, breathiness, asthenia (weakness), and strain (GRBAS) scale (for dysphonia) [22], and polysomnography (for obstruction of upper airways).

During selumetinib treatment, tumor-related deformity decreased from baseline in three patients and remained stable in one patient, motor dysfunction improved in two patients and remained stable in one patient, breathing difficulties related to severe obstructive sleep apnea syndrome completely resolved to a normal polysomnography, and dysphonia remained unchanged.

#### Statistical considerations

The SD response at C6 was positively correlated with SD at both C12 and the end of therapy (*r* = 0.758, *p* < 0.001 and 0.501, *p* < 0.001 respectively) but negatively correlated to PR/MR at C12 and at the end of follow up, (*r*=-0.766, *p* < 0.001 and *r*= -0.455, *p* < 0.001 respectively).

Moreover, the PR/MR response at C6 significantly, positively correlated with PR/MR at C12 and at the end of follow-up (*r* = 0.886, *p* < 0.001 and *r* = 0.554, *p* < 0.001 respectively), whereas PR/MR at C6 negatively correlated to SD at both 12 cycles and at the last observation (*r*=-0.772, *p* < 0.001 and *r*=-0.528, *p* < 0.001).

The PD response at C6 was correlated with the presence of worsening pain during therapy even after Bonferroni’s correction (*r*=-0,795, *p* < 0.001).

At C12, the PD response was significantly and positively correlated with PD at the end of follow-up (*r* = 0.717, *p* < 0.001) and was negatively correlated with CBR at the end of follow-up (*r*=-0.717, *p* < 0.001).

The SD at C12, after Bonferroni’s correction, correlated positively with SD and negatively with PR/MR at C12 ( *r* = 0.650, *p* < 0.001 and *r*=-0.490, *p* < 0.001 respectively), like that observed for SD at C6.

No correlation was found between basal volume, age at starting therapy, or sex and the type of response. Finally, no correlations were found by Pearson’s correlation analysis or Bonferroni’s correction regarding type and number of AEs, patient age at selumetinib initiation, timing of AE onset, or grade of AE severity.

## Discussion

Selumetinib has been shown to effectively shrink or stabilize the growth of PNs in patients with NF1. It can provide clinically meaningful benefits, such as pain relief and improved functional outcomes and health-related QoL [5, 10, 18, 23–30]. Since FDA approval in 2020 and EMA approval in 2021, this treatment is now available in more than 50 countries to manage inoperable and symptomatic PNs. As experience grows and several thousand patients are being treated, our study aims to add knowledge about the real-world use of selumetinib in children with NF1.

Research that used National Cancer Institute NF1 natural history data to assess the relationship between PN tumor volume, growth rate, and PN-related pain and functional morbidities, found that the majority of PNs were associated with PN-related morbidity at baseline, and that once PN-related symptoms develop, they are unlikely to resolve without treatment [31]. Given that clinical morbidities in patients with stable or growing PNs remain stable or worsen over time, the researchers concluded that any improvement observed in patients receiving therapy is likely treatment-related. They also found that large tumors were more likely to have associated motor dysfunction, and that rapid PN tumor growth was associated with an increased perception of pain requiring medication.

In our study, we highlight how the majority of cases were radiologically progressing at the time of starting therapy. This approach may reflect the compassionate use of the drug in the historical period analyzed. Sadly, due to the retrospective nature of the study, we do not have enough data to analyze an early CBR. Nevertheless, it emerges that at C24 a CBR was observed in 83% of cases. For the few published papers on the natural history of PNs, the lack of sex- and age-matched patient populations makes strong comparisons difficult. However, two main points emerge from the key papers on this topic. First, no PRs are reported in untreated PNs, and second, there are large variations in growth rate. As noted, we have a CBR at 24 cycles which coincides with the last observation.

The National Cancer Institute Natural History Study (NCT00924196) reported that 43% of patients had progressive disease (defined as ≥ 20% increase in tumor volume) over a median of 1.5–2 years. Compared to natural history studies where 30–63% of PNs progress ≥ 20% over similar or longer durations, our 17% progression rate at C24 seems to represent a substantial improvement. The benefit is particularly noteworthy, as natural history data show higher progression in younger patients, and our patients’ median age at onset of therapy.

Finally, at the last observation, a PR was observed in around one-third of patients and MR in 15%. The latter is a novel result that has never been explored for selumetinib so far. The median cycle of MR/PR was C12, ranging between 6 and 24 cycles. It may be worth keeping patients on therapy for at least 2 years when PD or serious AE are absent. The best PR/MR (independent of timepoint) was observed at C24 and corresponded to a 94% reduction from the baseline volume, representing the best response observed to date with selumetinib.

Our results suggest that the initial response, both at C6 and C12, potentially predicts the final outcome. Further prospective studies are desirable to confirm this preliminary finding.

Pain, social functioning, physical function impact, stigma, and emotional distress are the most important symptoms or concerns for patients with PNs [3]. Rapid PN tumor growth has been associated with increased pain [31] and pain to interfere with daily functioning [3].

In our study, at the initiation of selumetinib, pain was the most frequently reported symptom (51.4%), followed by deformity (35.7%), and motor dysfunction (15.7%), in line with the SPRINT study [5]. Selumetinib was associated with a significant reduction in pain perception between C6 and C48 (*p* < 0.05) independently by radiological response in favor of a clinical benefit. Pain outcomes for two patients in our study have been recently described in a case series [32]. The patients both had severe neuropathic pain at baseline, and both showed a marked reduction in or complete resolution of pain within a few weeks of selumetinib initiation, allowing analgesic therapy to be discontinued. We also observed pain regression, a clinical benefit, in patients with SD even as early as at C6, confirming the strong impact of selumetinib on pain, independently shown by significant radiological reduction of the lesion.

Selumetinib treatment has been reported to improve patient QoL [5]. Similarly, we observed a statistically significant improvement in patient-evaluated QoL. ECOG PS values were significantly improved with selumetinib treatment from baseline at all timepoints (C6 through C48; *p* < 0.05).

Selumetinib has reportedly also improved the functional prognosis of patients with inoperable PNs [33]. In our study, selumetinib was associated with decreased deformity in three out of four patients, improvement of motor dysfunction in two out of three, and resolution of breathing difficulty in the one patient who experienced this difficulty. In this regard, it is worth mentioning that evaluation of PN-related clinical signs and symptoms with standardized and repeatable tests can be challenging and time-consuming in clinical practice.

Previous studies of selumetinib in patients with PNs reported minor AEs (nausea, vomiting, diarrhea, asymptomatic CPK elevation, acneiform rash, and paronychia) and an acceptable safety profile in long-term use among pediatric populations [5, 24, 25, 34, 35]. The most common AEs involved the skin [18, 36]. As with previous studies, most AEs in our study were of grade 1 or 2 intensity (78.0%) and manageable. The most common AEs were dermatitis, paronychia, xerosis with or without itching, increases in CPK levels, and hair depigmentation, revealing no surprises in AEs or their management. Treatment was suspended in three patients due to AEs. The early onset and number of AEs were independent of the patients’ sex or age at selumetinib initiation.

One of the patients who had undergone a biopsy before initiating treatment with selumetinib was an 11-year-old boy diagnosed with PN on his leg. The patient achieved MR at C6 and PR at C12 after treatment initiation. However, at C18, the PN tumor volume increased almost to the initial levels and the patient died due to an MPNST progression.

Another patient was treated with selumetinib for 42 cycles and had a robust response in terms of pain control, SD until C36, at which timepoint PD was observed and confirmed at C42. Consequently, with no radiologic signs of malignancy, selumetinib was suspended (Supplementary Tables 1 and 2).

We acknowledge that not all data was available for all patients, and other study limitations deserve mention. First, data on the radiologic behavior of target PNs prior to selumetinib initiation were not available and, although around 60% of cases were experiencing a growing lesion before the therapy, thorough data on volumetric changes are lacking, which could influence any kind of interpretation of the PFS rate. Second, patients need to undergo functional evaluations and QoL assessments every 6 months during the first 2 years of treatment; unfortunately, in our study, not all evaluation parameters were collected accurately. Future prospective studies with longer follow-up and standardized functional assessments will be essential to further validate these findings and refine clinical management strategies.

In our study, all relevant endpoints related to clinical improvements were evaluated. A key innovation of this study is the assessment of clinical benefits independently by the radiological response, of a CBR at 24 months, and the evaluation of a major response recognizing a reduction of more than 50% of the basal volume of lesions. Existing literature demonstrates that patients achieving SD still benefit from treatment, as it prevents tumor progression and alleviates symptoms; we demonstrated a significant reduction in PN-related symptoms, particularly pain, independently by radiological reduction, highlighting the chance of an analgesic pleiotropic effect of the selumetinib. Furthermore, continued therapy beyond SD leads to sustained pain reduction, supporting the recommendation to maintain standard high-dose treatment whenever feasible. We believe that CBR better defines specific potential benefits of selumetinib and should be a further object of evaluation in future prospective studies.

## Conclusion

We present novel data of a correlation between early radiological response (at C6) and the response at the end of observation, a clinical benefit in terms of pain control in stable lesions, CBR at C24 confirming the favorable course of treated patients and the evaluation of major responses. Further studies are warranted to refine our understanding of tumor growth dynamics, optimal treatment strategies and clinical benefit assessment beside and beyond the radiological tumor response. If confirmed, these results could have significant clinical implications for the management of NF1-associated PNs.

## Supplementary Information

Below is the link to the electronic supplementary material.

Supplementary Material 1

## Data Availability

The data presented in this study are available on request from the corresponding author, but access is restricted due to privacy concerns.
