# Supplementary material for: Real-world experience with selumetinib in children with neurofibromatosis type 1: a multicentric retrospective study
Source: J Neurooncol. 2025 Aug 13;175(3):1027–37. doi: 10.1007/s11060-025-05197-5 (PMC12511227; doi:10.1007/s11060-025-05197-5)
Supplement: Supplementary file 1 — Supplementary Material 1 [file 11060_2025_5197_MOESM1_ESM.doc]

**Real-world experience with selumetinib in children with neurofibromatosis type 1: a multicentric retrospective study**

**Journal of Neuro-Oncology**

**Authors:** Claudia Santoro, Mariachiara Servedio, Maria Cristina Diana, Irene Russo, Elena Arkhangelskaya, Gianluca Piccolo, Andrea Santangelo, Angela Mastronuzzi, Antonella Cacchione, Maya El Hachem, Carmela Russo, Mario Cirillo, Ilaria Cecconi, Antonio Grasso, Mariateresa Loiotine, Nicola Santoro, Mariachiara Resta, Carmela De Meco, Consolata Soddu, Eugenia Spreafico, Bartolomeo Rossi, Chiara Fossati, Chiara Leoni, Silverio Perrotta, Teresa Perillo

**Corresponding author:** Claudia Santoro

Department of Women’s and Children’s Health and General and Specialized Surgery, University of Campania “Luigi Vanvitelli”, Naples, Italy.
Tel.: 039-0815665454; Email: [claudia.santoro@unicampania.it](mailto:claudia.santoro@unicampania.it)

**Supplementary material**

# Supplementary Table 1.

Demographic and clinical characteristics at end of follow-up for 9 patients with PNs treated with selumetinib

| **Patient ID** | **Sex** | **PN anatomical region** | **Biopsy or surgery prior to selumetinib initiation** | **Other pharmacotherapies, including PN-targeted** | **PN symptom(s)** | **Reason for therapy suspension** | **Discontinued therapy** | **Duration of therapy**  **(years)** |
| --- | --- | --- | --- | --- | --- | --- | --- | --- |
| **1** | F | Thigh | Suspected malignant evolution | NO | Motor dysfunction | Radiologic progression | YES | 3.4 |
| **2** | F | Pelvis | Suspected malignant evolution | NO | Pain and motor dysfunction | Radiologic progression | YES | 2.8 |
| **3** | M | Thigh |  | NO | Pain | COVID-19 pandemic | NO | 3.0 |
| **4** | M | Pelvis |  | NO | Pain |  | NO | 4.8 |
| **5** | F | Neck | Suspected malignant evolution | Imatinib 11/2013–03/2014 Pegylated interferon 10/2014–11/2015 | Pain and disfigurement | Progression, MPNST, diseased patient | YES | 1.8 |
| **6** | M | Leg | Suspected malignant evolution | Imatinib 02/09/2012–01/10/2014 Pegylated interferon 01/11/2015–08/12/2016 | Pain, limp and dysmetria | Progression, MPNST, diseased patient | YES | 1.8 |
| **7** | M | Thorax |  | NO | Pain |  | NO | 4.8 |
| **8** | F | Flank |  | NO | Itching and discomfort |  | NO | 2.0 |
| **9** | M | Tongue |  | NO | Speech impairment and bleeding |  | NO | 1.9 |

F, female; ID, identification; M, male; PN, plexiform neurofibroma; MPNST, malignant peripheral nerve sheath tumor.

# Supplementary Table 2.

Changes from baseline in tumor volumes and treatment-related responses at the last observation for 9 patients with plexiform neurofibromas treated with selumetinib

| **Patient ID** | **Volume at T0 (mL)** | **Vol**u**me at C6 (mL)** | **Response at C6** | **Volume at C12 (mL)** | **Response at C12** | **Volume at C18 (mL)** | **Response at C18** | **Volume at C24 (mL)** | **Response at C24** | **Volume at C30 (mL)** | **Response at C30** | **Volume at C36 (mL)** | **Response at C36** | **Volume at C42 (mL)** | **Response at C42** |
| --- | --- | --- | --- | --- | --- | --- | --- | --- | --- | --- | --- | --- | --- | --- | --- |
| **1** | 100.00 | 89 | SD | 96.00 | SD | 95.00 | SD | 115 | SD | 117 | SD | 137 | PD | 161 | PD |
| **2** | 26.50 | NA | NA | 29.00 | SD | 27.00 | SD | 29.5 | SD | 28.5 | SD | 45.7 | PD | 72.452 | PD |
| **3** | 495.00 | 546 | SD | 693.00 | PD | 836.55 | PD | 836 | PD |  |  |  |  |  |  |
| **4** | 0.71 | NA | NA | 1.60 | PD | 1.10 | PD |  |  |  |  |  |  |  |  |
| **5** | 59.00 | 204 | PD | 204.00 | PD | 250.00 | PD |  |  |  |  |  |  |  |  |
| **6** | 438.00 | 318 | PR | 254.00 | PR | 580.00 | PD |  |  |  |  |  |  |  |  |
| **7** | 126.36 | NA | NA | 125.60 | SD |  |  | 166.45 | PD |  |  |  |  |  |  |
| **8** | 300.30 | NA | NA | 374.40 | PD |  |  | 534.9 | PD |  |  |  |  |  |  |
| **9** | 3.90 | NA | NA | 5.80 | PD | 3.70 | SD | 5.67 | PD |  |  |  |  |  |  |

C, cycle; ID, identification; NA, not available; PD, progressive disease; PR, partial response; SD, stable disease; T0, time 0, within 1 month prior to starting therapy.

# Supplementary Fig. S1

A schematic representation of radiological response observed in a subgroup of patients who had a volumetric assay at C6. The figure also shows their outcome at the end of follow-up


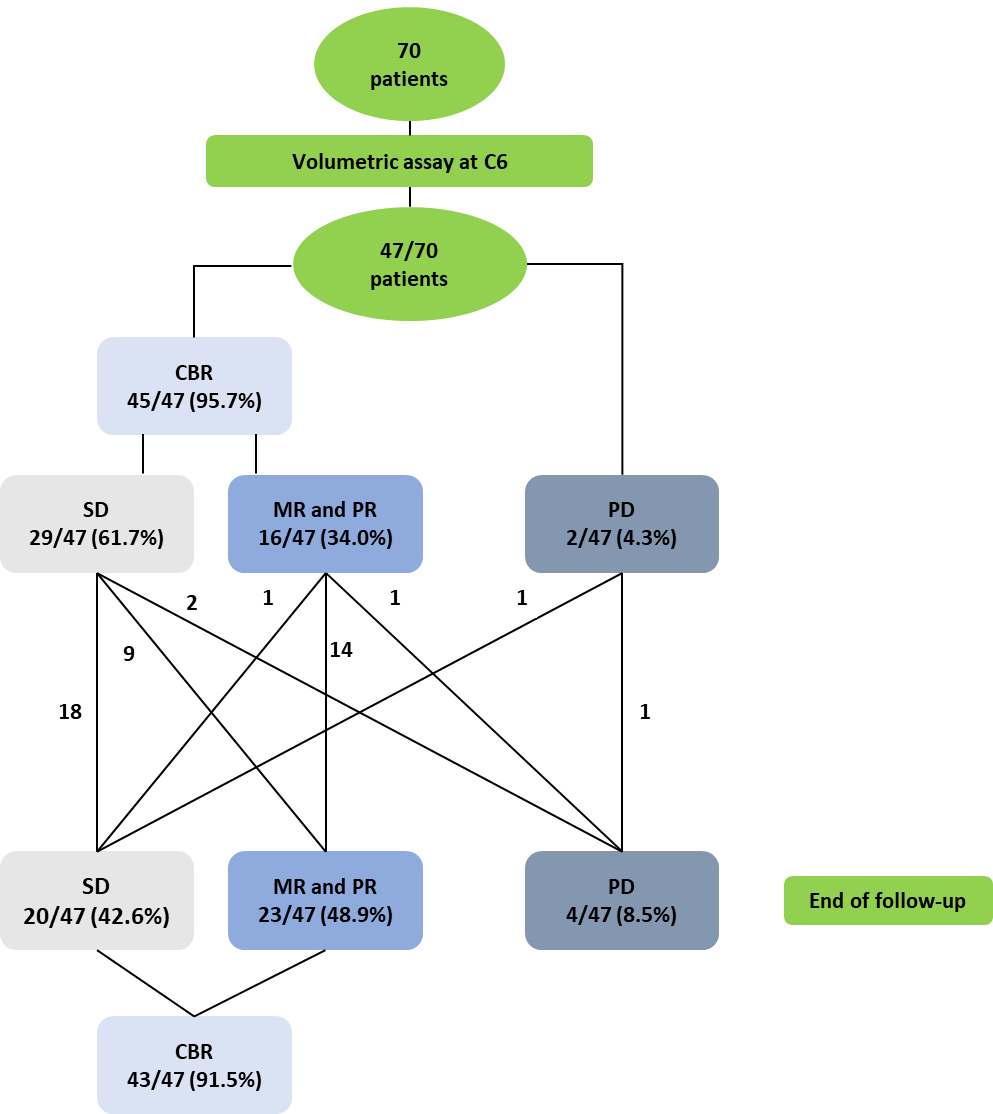


C, cycle; CBR, clinical benefit rate; MR, major response; PR, partial response; SD, stable disease.

# Supplementary Fig. S2

Percentage change in target PN volume over time


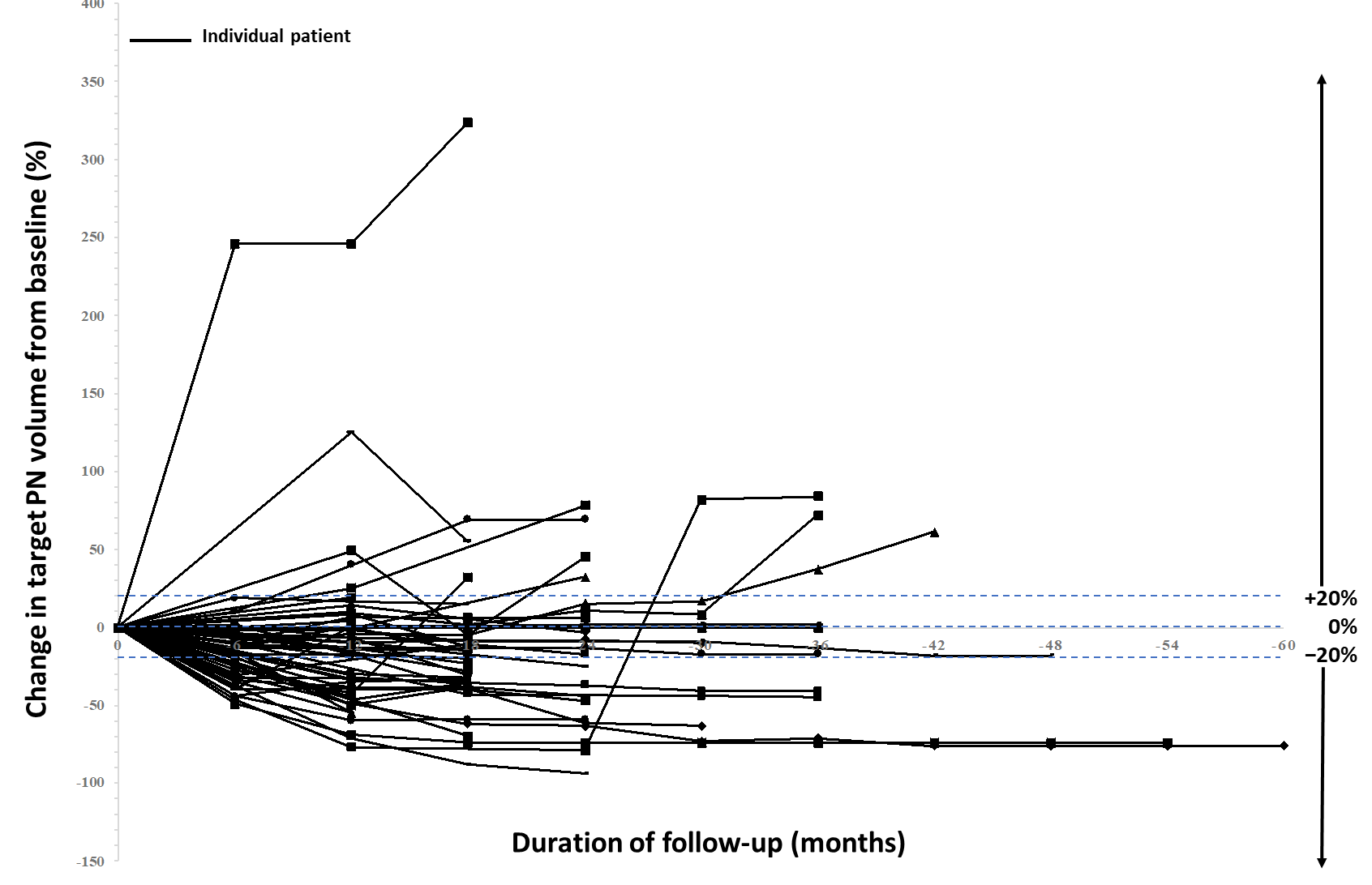


PN, plexiform neurofibroma.
